# Elevated Levels of FMR1 mRNA in Granulosa Cells Are Associated with Low Ovarian Reserve in FMR1 Premutation Carriers

**DOI:** 10.1371/journal.pone.0105121

**Published:** 2014-08-25

**Authors:** Shai E. Elizur, Oshrit Lebovitz, Sanaz Derech-Haim, Olga Dratviman-Storobinsky, Baruch Feldman, Jehoshua Dor, Raoul Orvieto, Yoram Cohen

**Affiliations:** Department of Obstetrics and Gynecology, Chaim Sheba Medical Center, (Tel Hashomer), Ramat Gan, Israel, and Sackler Faculty of Medicine, Tel Aviv University, Tel Aviv, Israel; CNRS UMR7275, France

## Abstract

**Aim:**

To assess the role of mRNA accumulation in granulosa cells as the cause of low ovarian response among FMR1 premutation carriers undergoing pre-implantation genetic diagnosis (PGD).

**Design:**

Case control study in an academic IVF unit. Twenty-one consecutive FMR1 premutation carriers and 15 control women were included. After oocyte retrieval the granulosa cells mRNA levels of FMR1 was measured using RT-PCR.

**Results:**

In FMR1 premutation carriers, there was a significant non-linear association between the number of CGG repeats and the number of retrieved oocytes (p<0.0001) and a trend to granulosa cells FMR1 mRNA levels (p = 0.07). The lowest number of retrieved oocytes and the highest level of mRNA were seen in women with mid-size CGG repeats (80–120). A significant negative linear correlation was observed between the granulosa cells FMR1 mRNA levels and the number of retrieved oocytes (R^2^ linear = 0.231, P = 0.02).

**Conclusion:**

We suggest that there is a no-linear association between the number of CGG repeats and ovarian function, resulting from an increased granulosa cells FMR1 mRNA accumulation in FMR1 carriers in the mid-range (80–120 repeats).

## Introduction

Fragile X Syndrome (FXS), the most common form of inherited mental retardation, is caused by a trinucleotide repeat expansion (CGG) in the 5′-untranslated region of the fragile X mental retardation 1 (FMR1) gene located at Xq27.3. Patients with fragile X–related mental retardation, carry the full mutation CGG-repeat expansions (>200 repeats), which are generally accompanied by transcriptional silencing of the FMR1 gene, and consequent absence of the encoded *FMR1* protein (FMRP) [1].

The mutation tends to expand in size as it is passed from mother to offspring. While premutation alleles (n = 55–200) can expand to a full mutation within one generation, the intermediate repeat length (45–54 repeats) was defined based on the ability to expand to a full mutation after two or more generations [2]. The prevalence of premutation alleles is 1 in 350 females, and the prevalence of intermediate alleles is about 6% of females [3].

Amplification of the CGG triplet number above the normal range (n = 5–44) towards the so-called premutation status (n = 55–200) is associated with increased risk for fragile X-associated premature ovarian insufficiency (POI) in females [4], [5], and fragile X-associated tremor/ataxia syndrome (FXTAS) in males.

FXTAS is a neurodegenerative disorder caused by aberrant expansion of CGG repeats in 5′ UTR of *FMR1* gene. The characterized clinical features of FXTAS are progressive cerebellar gait ataxia, intention tremor, cognitive decline and some psychiatric involvement [6]. Premutation-length CGG repeats induced RNA toxic gain-of-function, and the accumulation of elevated FMR1 mRNA is believed to be an important and proximal event in the pathogenesis of FXTAS.

POI (defined by cessation of menses prior to age 40) affects approximately 1% of reproductive-age female population. While in over half of cases, no etiology can be identified, recent studies suggest that both the premutation and the intermediate repeat length are associated with overt POI [7], [8]. Carriers for the CGG premutation on one allele have a high risk (16–26%) for POI [9], compared with only 1% of females in the general population and enter menopause earlier (approximately 5 years) than non-carrier [10]. Moreover, premutation carriers have impaired ovarian function, as evident by abnormal ovarian reserve biomarkers (serum anti-mullerian hormone, FSH, inhibin B, inhibin A and progesterone) and a reduced ovarian response to controlled ovarian hyperstimulation (COH), resulting in higher gonadotropin dosages and fewer embryos [11]–[17].

Previous reports suggest that there is a non-linear association between the number of CGG repeats and ovarian function. Allen et al. and Ennis et al. [18], [19] found a non-linear association of decreased reproductive lifespan and menopause age with premutation size. The mid-range repeat size group (80–100 repeats), not the lowest or highest groups, had an increased risk for ovarian insufficiency. Bibi et al. [17] found that premutation carriers with <100 CGG repeats suffer from impaired ovarian response and decreased fertilization rate during in-vitro fertilization (IVF) treatment compared to patients with ≥100 CGG repeats. However, they were not able to compare these variables among the 80–100 CGG size group separately. In Wittenberger et al. [5] comprehensive review, they summarized this nonlinear relationship, and concluded that the risk of POI increases with increasing number of CGG repeat size between 59 and 99, thereafter the risk plateaus or even decreases.

Although the underlying molecular mechanism of POI is not clear, consistent features are that the full mutation females, with transcriptional silencing of the FMR1 gene, and absence of FMRP, are not affected with POI, while females with premutation alleles show normal or slightly reduced levels of FMRP and elevated FMR1 mRNA levels [20]. This observation led to the hypothesis that accumulation of FMR1 mRNA is toxic to the ovary and is responsible to POI in a similar mechanism to the FXATS RNA gain-of-function toxicity.

Prompted by the aforementioned observations we aimed to explore the molecular basis of ovarian insufficiency in women with premutation in FMR1 gene, by measuring the FMR1 mRNA levels in patients undergoing COH for IVF and evaluating their correlation to COH variables.

## Materials and Methods

### Patients and IVF treatment

This study was approved by the Institutional Ethical Review Board of Sheba Medical Center, Israel. All patients that were included in this study signed a written informed consent.

In Israel, as part of a national prenatal screening program, all women who wish to conceive are advised to determine their FMR1 CGG carrier status. All FMR1 premutation carriers (55–200 CGG repeats) are further referred to a genetic consultation to consider in vitro fertilization (IVF) and pre-implantation genetic diagnosis (PGD) in order to avoid the risk of CGG expansion in offspring. Therefore, in all women undergoing IVF in our center the CGG repeat status is known (either FMR1 carriers (55–200 CGG repeats) or normal <55 CGG repeats).

The study population consisted of all consecutive FMR1 premutation carriers referred to our IVF unit for IVF-PGD treatment, during a 18 month period, who reached the ovum pick-up (OPU) stage. The control group consists of patients, with less than 55 CGG repeats, undergoing IVF-ICSI for male factor infertility, matched by age, treated in the same period. The study required no modification of our routine IVF protocols. Data on patients' age, infertility-treatment-related variables and ovarian stimulation characteristics, number of oocytes retrieved, and number of embryos transferred per cycle were collected and compared between the two study groups.

The selection of type of COH protocol used was the decision of the treating physician. In all protocols, gonadotropins were administered in variable doses, depending on patient age and/or ovarian responsiveness in previous cycles, and further adjusted according to serum estradiol (E_2_) levels and vaginal ultrasound measurements of follicular diameter obtained every 2 or 3 days.

Thirty four to 36 h after HCG injection, oocytes were aspirated by the ultrasound guided transvaginal route and the pooled follicular fluids containing granulosa-cells were collected from each patient.

### Retrieval of cumulus cells

After oocyte retrieval mural granulosa cells from follicular fluid were washed with PBSX1 to remove the residual blood and were stored at −80°C until RNA extraction.

### RNA isolation, RT-PCR and qRT-PCR

Total RNA isolation was performed using the Quick-RNA™ Microprep Kit (ZYMO RESEARCH, USA) according to the manufacturer's instructions to significantly reduce contamination from both genomic DNA and proteins. The RNA quality was assessed using a NanoDrop ND-1000 Spectrophotometer. RNA (100–500 ng) was transcribed to generate cDNA using qScript cDNA Synthesis Kit (Quanta BioSciences) with optimized blend of random hexamers and oligo(dT) primers according to the manufacturer's instructions. Real-time quantitative PCR was performed using the StepOnePlus™ System(Applied Biosystems). The mRNA levels of genes were measured by a PerfeCTa SYBR Green FastMix (Quanta BioSciences) according to the manufacturer's instructions, and the human Hypoxanthine Guanine Phosphoribosyltransferase was used as a control housekeeping gene. Melting curve analysis was performed to confirm amplification of specific transcripts. Each reaction was run in triplicate and in parallel. The expression levels of transcripts were calculated by the relative quantification (ΔΔ*Ct*) study method by using SDS software (Applied Biosystems).

### Primer sequences used for amplifications were as follows:

h-FMR1_F: 5′-AACAAAGGACAGCATCGCTAATG-3′

h-FMR1_R: 5′-CAAACGCAACTGGTCTACTTCCT-3′

H-HPRT_F: 5′-AGATGGTCAAGGTCGCAAGCT-3′

H-HPRT_R: 5′-TCAAATCCAACAAAGTCTGGCTTA-3′

### DNA extraction and conversion by bisulfite modification

Isolation of DNA and RNA from granulosa cells was performed using the AllPrep DNA/RNA Micro kit (Qiagen, Valencia, CA) according to the AllPrep DNA/RNA protocol. The EZ DNA Methylation-Gold kit was used for bisulfite treatment of genomic DNA according to the manufacturer's instructions (ZYMO Research).

### Quantitative Methylation-Specific PCR (qMSP)

QMSP was performed as described previously (21). Briefly, multiplex quantitative methylation-specific PCR amplification was performed on the StepOnePlus™ Real-time PCR System (Applied Biosystems), using bisulfite-treated DNA as template, amplification primers designed to avoid CpG dinucleotides in the sense strand within the promoter of the FMR1 gene, probes for unmethylated and methylated copies of the FMR1 gene and qPCR BIO Probe Mix Hi-Rox PCR master mix (PCR Bio systems).

The primer concentrations were 1 µM and the probe concentrations were 150 nM. The PCR conditions were an initial 95°C denaturation for 2 min followed by amplification cycles consisting of 95°C for 5 s and 60°C for 20 s for 40 cycles.

### qMSP primers and probes sequences were as follows:

FMR1 amplification primers*:

FMR1F: GYGTTTTTAGGTTATTTGAAGAGAGAGGG

FMR1R: CRACCCRCTACRAATATAAACACTAAAACC

(Y = T or C; R = G or A).

TaqMan probes*:

FMR1M (methylation specific probe): CGGGGTCGAGGGGTTGAGTTCGCG

FMR1UM (non-methylation probe): TGGGGTTGAGGGGTTGAGTTTGTGGG

The 5′-ends were labeled with FAM (methylation specific probe) and HEX (unmethylated DNA probe) and were quenched by the addition of Black Hole Quencher 1 to the 3′ end of the oligonucleotide.

*- Designed by Coffee et al [21]

### Statistical analysis

The statistical analysis was performed using Student t test, Mann-Whitney, chi-squared test, Pearson correlation and polynomial regression analysis as appropriate. The differences among groups were considered significant when the p-value was <0.05.

## Results

Twenty-one consecutive FMR1 premutation carriers and 15 control women were evaluated. There were no differences between the two groups regarding patients' age, parity, duration of stimulation, number of embryos transferred or pregnancy rates (Table 1). As expected, the premutation carriers group had a significantly higher basal FSH/LH ratio, used significantly higher gonadotropins dosage during ovarian stimulation and had a lower number of retrieved oocytes (Table 1).

**Table 1:** Clinical and laboratory characteristics of the study (carriers of FMR1 premutation) and control groups.

	FMR1 Premutation	Control	
	N = 21	N = 15	P value
**Age (mean) (SD)**	31.5 (3.4)	30.8 (4.3)	ns
**Parity (median)**	0	0	ns
**Mean FSH (IU) (basal) (SD)**	8.2 (2.0)	7.0 (1.7)	0.08
**Mean LH (IU) (basal) (SD)**	3.7 (1.7)	4.9 (1.9)	ns
**Mean Basal FSH/LH ratio (SD)**	2.4 (1.3)	1.4 (0.7)	0.01
**Mean Estradiol (basal) (pmol/L) (SD)**	152 (58)	156 (87)	ns
**Mean Total Gonadotropins used in stimulation (IU) (SD)**	2588 (1198)	1865 (990)	0.04
**Mean duration of stimulation (days) (SD)**	10.8 (2.8)	10 (1.9)	ns
**Mean peak estradiol (pmol/L) (SD)**	6399 (3347)	8470 (2508)	0.06
**Mean no. oocyte retrieved (SD)**	9 (7.1)	13.1 (5.7)	0.02
**Mean no. embryo transferred (SD)**	1.4 (1.2)	1.7 (0.7)	ns
**No. of pregnancies**	4	2	ns
**Mean FMR1 repeats (range)**	102 (64–200)	<55	

**Figure 1:**
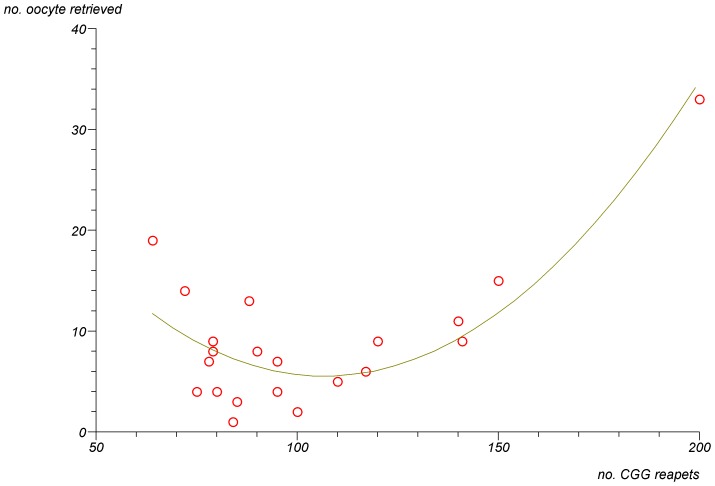
A non-linear association between the number of retrieved oocytes during IVF cycle and the number of CGG repeats in FMR1 premutation carriers (55–200 repeats). (P<0.0001).

**Figure 2:**
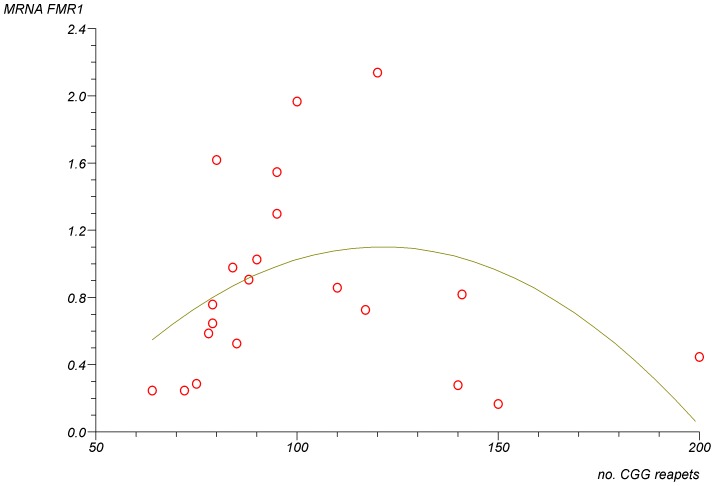
A trend for a non-linear association between FMR1 mRNA levels in granulosa cells of FMR1 premutation carriers (55–200 repeats) and the number of CGG repeats. (P = 0.07).

**Figure 3:**
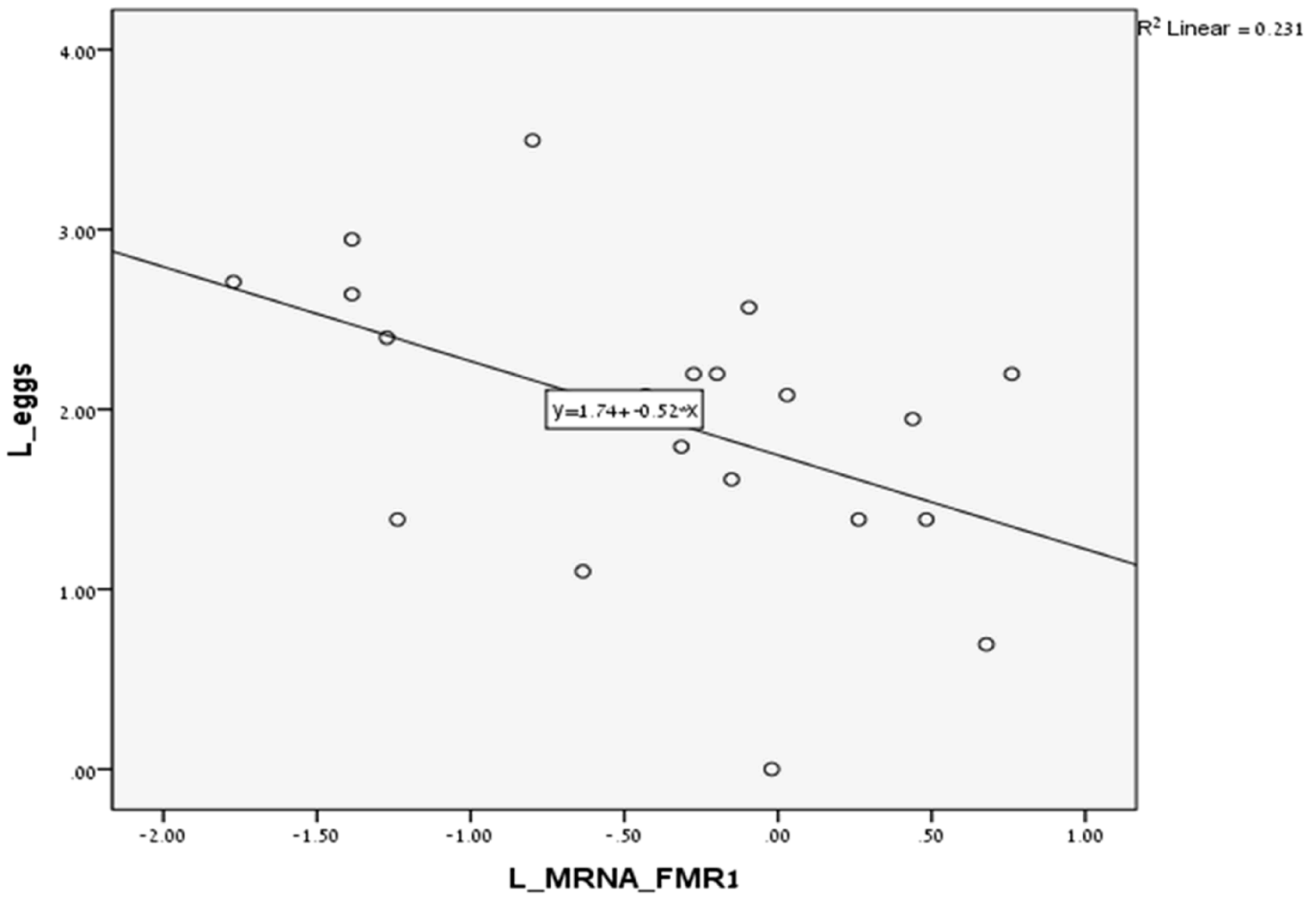
The number of retrieved oocyte during IVF cycle according to mRNA levels of FMR1 in granulosa cells in FMR1 premutation carriers (55–200 repeats). (R^2^ linear = 0.231, P = 0.02).

In FMR1 premutation carriers there was a significant non-linear association between the number of CGG repeats and the number of oocyte retrieved (Fig. 1) (p<0.0001). The lowest number of oocytes was retrieved in women with mid-size CGG repeats (80–120). In addition, there was a trend for a non-linear association between the number of CGG repeats and the levels of FMR1 mRNA levels in granulosa cells (Fig. 2) (p = 0.07). The highest level of mRNA was seen in women with mid-size CGG repeats (80–120). Finally, a significant negative linear correlation was observed between the FMR1 mRNA levels in granulosa cells and the number of oocytes retrieved (Fig. 3) (R^2^ linear = 0.231, p = 0.02). We further analysed the group of women with FMR1 premutation according to the number of CGG repeats. We compared women with mid-size GGG repeats (80–120) to premutation carriers' with lower or higher number of CGG repeats. Women with mid-size GGG repeats had a significantly lower number of retrieved oocytes (5.6±3.5) compare to premutation carriers' with lower or higher number of CGG repeats (13.5±8.5) (p = 0.004). Moreover, the FMR1 mRNA level in granulosa cells was significantly higher in women with mid-size GGG repeats (1.7±0.5 units) compared to premutation carriers' with lower or higher number of CGG repeats (0.9±0.4, p = 0.005) and women with normal CGG repeats (1.1±0.3, p = 0.02). These findings suggest that high mRNA levels in granulosa cells might be the reason for low ovarian reserve in women with mid-size GGG repeats (80–120).

**Figure 4:**
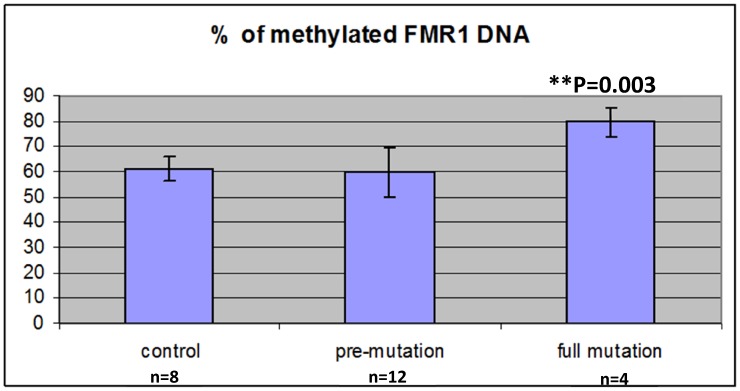
Methylated FMR1 DNA (%) in granulosa cells in women with normal CGG repeats (<55), premutation carriers (55–200) and full mutation (>200).

**Figure 5:**
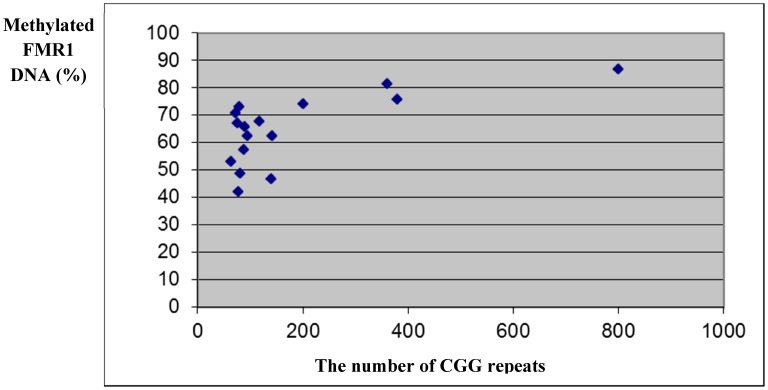
Methylated FMR1 DNA (%) in granulosa cells according to the number of CGG repeats in premutation carriers (55–200) and full mutation (>200).

In order to find out whether the elevated levels of FMR1 mRNA in mid-size premutation carriers are due to different FMR1 methylation status, we evaluated the methylation status of the FMR1 promoter region in DNA extracted from granulosa cells obtained from women with normal CGG length (<55 CGG repeats), premutation carriers (55–200 repeats) and full mutation (>200 repeats). As expected, women with full mutation had significantly higher DNA FMR1 methylation (79.5%) compared to premutation carriers (59.7%) and controls (61.2%) (p = 0.003) (Fig. 4). In premutation carriers no association was observed between the number of CGG repeats, levels of FMR1 mRNA and DNA FMR1 methylation. Only in women with 200 CGG repeats and more, we observed significantly higher percentage of FMR1 DNA methylation (Fig. 5), and, in turn, reduced levels of FMR1 mRNA (0.47±0.56 units).

## Discussion

To the best of our knowledge, this is the first study to date suggesting that mRNA mediated toxicity in granulosa cells might be the pathogenesis for ovarian insufficiency in women with FMR1 premutation.

As in previous studies [11]–[17] our study also observed that in FMR1 premutation carriers there is a significant impairment in ovarian reserve biomarkers, such as high basal FSH levels and FSH/LH ratio [22], as well as a reduced response to COH protocol (Table 1). In addition, as in previous studies [18], [19] our study demonstrated a significant non-linear association between the number of CGG repeats and ovarian function with the mid-size range (80–120) having the worst prognosis. In the present study, women with 80–120 CGG repeats had significantly less oocyte retrieved compared to women with higher and lower repeats (Fig. 1). Moreover, we were able to demonstrate that there is also a significantly non- linear association between the number of CGG repeats and the levels of FMR1 mRNA in granulosa cells (Fig. 2). Women in the mid- range CGG repeats (80–120) had significantly higher levels of FMR1 mRNA levels in granulosa cells compared to women with higher and lower repeats and these levels were significantly negatively correlated to the number of oocyte retrieved (Fig. 3) (R^2^ linear = 0.231, p = 0.02). These findings strongly suggest that FMR1 mRNA accumulation might be the cause of ovarian insufficiency in women with premutation in the FMR1gene.

Elevated levels of FMR1 mRNA in premutation carriers do not seem to be due to changes in FMR1 methylation status in the granulosa cells. We observed a similar methylation status of the FMR1 promoter region in premutation carriers and women with normal number of CGG repeats. As expected, only women with full mutation had increased levels of FMR1 methylation and reduced FMR1 mRNA levels, compared to premutation carriers and controls (Fig. 4,5).

Currently, the mechanism of the impaired ovarian function related to the FMR1 premutation is unknown. A low initial ovarian follicles pool, an accelerated rate of atresia, or any etiology that might impair follicle function, may all be responsible for the continuum impairment of ovarian function in women with FMR1 premutation.

In premutation male carriers having FXTAS an mRNA gain-of-function toxicity mechanism has been suggested for the pathogenesis [23]. This type of RNA gain-of function mechanism has been suggested as a mechanism for triplet repeat-related ataxias, such as spinocerebellar ataxia (SCA) 8, SCA10, and SCA12, and for myotonic dystrophy (DM) [24].

FXTAS has been suggested to be caused by titration of RNA-binding proteins by the expanded CGG repeats. It has been shown that mRNAs containing expanded CGG repeats form large and dynamic intranuclear RNA aggregates that recruit several RNA-binding proteins among them Sam68 that is sequestered by expanded CGG-repeat aggregates and thereby loses its splicing-regulatory function. As a consequence, Sam68-regulated splicing is altered in FXTAS patients [25]. Moreover, it has been previously shown that Sam68^−^/^−^ females mice are subfertile and display a dramatic reduction in the cumulative number of pups delivered during their lifespan. Sam68 directly binds the mRNAs for the follicle-stimulating hormone (FSH) and the luteinizing hormone receptors (FSHr and LhCGr), which were down regulated in ovaries of adult knockout females [26]. Therefore, defects in such protein can strongly reduce ovarian response to gonadotropins exactly as shown in FMR1 premutation carriers.

In conclusion, our study supports previous findings showing that FMR1 premutation carriers have diminished ovarian reserve and alerted response to gonadotropins. We suggest that there is a no-linear association between the number of CGG repeats and ovarian function and this is due to increased FMR1 mRNA accumulation in granulosa cells in FMR1 carriers in the mid-range (80–120 repeats).

Further studies are required to better understand the mechanism of mRNA toxicity in the ovaries of FMR1 premutation carriers.
